# R848 Is Involved in the Antibacterial Immune Response of Golden Pompano (*Trachinotus ovatus*) Through TLR7/8-MyD88-NF-κB-Signaling Pathway

**DOI:** 10.3389/fimmu.2020.617522

**Published:** 2021-01-18

**Authors:** Yongcan Zhou, Xiaojuan Chen, Zhenjie Cao, Jianlong Li, Hao Long, Ying Wu, Zhengshi Zhang, Yun Sun

**Affiliations:** ^1^State Key Laboratory of Marine Resource Utilization in South China Sea, Hainan University, Haikou, China; ^2^Hainan Provincial Key Laboratory for Tropical Hydrobiology and Biotechnology, College of Marine Science, Hainan University, Haikou, China

**Keywords:** R848, TLR7/8, antibacterial immune response, Myd88, NF-κB

## Abstract

R848 is an imidazoquinoline compound that is a specific activator of toll-like receptor (TLR) 7/8 and is often used in immunological research in mammals and teleosts. However, the immune responses initiated by R848 through the TLR7/8 pathway in response to bacterial infection remain largely unexplored in teleosts. In the current study, we investigated the antibacterial response and the participating signaling pathway initiated by R848 in golden pompano (*Trachinotus ovatus*). We found that R848 could stimulate the proliferation of head kidney lymphocytes (HKLs) in a dose-dependent manner, enhance the survival rate of HKLs, and inhibit the replication of bacteria *in vivo*. However, these effects induced by R848 were significantly reduced when chloroquine (CQ) was used to blocked endosomal acidification. Additionally, an *in vivo* study showed that R848 strengthened the antibacterial immunity of fish through a TLR7/8 and Myd88-dependent signaling pathway. A cellular experiment showed that Pepinh-MYD (a Myd88 inhibitor) significantly reduced the R848*-*mediated proliferation and survival of HKLs. Luciferase activity analysis showed that R848 enhanced the nuclear factor kappa B (NF-κB) activity, whereas this activity was reduced when CQ and Pepinh-MYD were present. Additionally, when an NF-κB inhibitor was present, the R848-mediated pro-proliferative and pro-survival effects on HKLs were significantly diminished. An *in vivo* study showed that knockdown of TLR7, TLR8, and Myd88 expression in golden pompano *via* siRNA following injection of R848 resulted in increased bacterial dissemination and colonization in fish tissues compared to that of fish injection of R848 alone, suggesting that R848-induced antibacterial immunity was significantly reduced. In conclusion, these results indicate that R848 plays an essential role in the antibacterial immunity of golden pompano *via* the TLR7/8-Myd88-NF-κB- signaling pathway.

## Introduction

R848 (Resiquimod) is an immune response modifier that is also known for its potential antiviral activity (1, 2). Clinical studies have shown that this low-molecular-weight compound can be successfully used in the treatment of infectious viral diseases caused by hepatitis C virus or the herpes simplex virus (1–4). R848 affects antiviral activity by reducing viral shedding and viral reoccurrence (1–4). R848 is recognized by toll-like receptor (TLR) 7 and TLR8, synergistically activates immune cells, and induces an environment of T helper (Th) 1 immune responses (5–8). It acts as a selective activating ligand for both TLR7 and TLR8 in humans but only TLR7 in mice (9, 10). R848 activates TLR7/TLR8 in a Myd88-dependent signaling pathway, then activates transcription factors NF-κB (nuclear factor kappa B) and JNK (c-Jun NH2-terminal kinase) and ultimately induces the production of Th1 cytokines, including IFN I (type I interferon), IL (interleukin)-6, IL-12, IFN-γ, TNF-α (tumor necrosis factor-α), and inhibits the expression and secretion of the Th2 cytokine IL-4 (11–14). In addition, R848 activates pDCs (plasmacytoid dendritic cells), monocytes, and macrophages through TLR7/8 to secrete a variety of cytokines that mediate innate and acquired immunity (3–6).

TLR7 and TLR8 are highly conserved in vertebrates, exhibiting similar structures, including 13-15 LRR (leucine rich repeat) domains among mammals. Previous studies found that the loci of both TLR7 and TLR8 are located on the same chromosome in fish and mammals (15–17). TLR7 and TLR8 are expressed in endosomal compartments but not on the cell surface, such as the ER (endoplasmic reticulum), endolysosomes, lysosomes, and endosomes (18, 19). TLR7 and TLR8 are responsible for recognizing viruses, ssRNA (synthetic single-stranded RNA), and some imidazoquinoline compounds, such as R848 and R837, and inducing antiviral immune responses (20, 21). In addition, recent studies show that TLR7 and TLR8 can also recognize bacteria leading to antibacterial immunity (22). To date, TLR7 has been identified in many teleosts, including zebrafish (*Danio rerio*) (23), Japanese flounder (*Paralichthys olivaceus*) (24), channel catfish *(Ictalurus punctatus*) (25), common carp (*Cyprinus carpio*) (26), rainbow trout (*Oncorhynchus mykiss*) (15), and sea bream (*Sparus aurata*) (27). TLR8 has been identified in zebrafish (23), channel catfish (25), rainbow trout (15), sea bream (27), Atlantic salmon (*Salmo salar*) (28), and many other species as well. In some of the fish listed above, TLR7 and TLR8 have been shown to respond to viruses or ssRNA (29–31). Therefore, TLR7 and TLR8 play important roles in host immunity to viral infections in fish. As an agonist of TLR7/TLR8, R848 has been reported to induce immune responses in many mammals (32, 33). However, little is known about the immune function induced by R848 and associated working mechanism in teleosts, especially the antibacterial immunity.

Golden pompano (*Trachinotus ovatus*) is an economically important fish with desirable flavor and high nutritive value and is also the primary species raised in offshore cages in China (34). In this study, golden pompano was used as a model, and the antibacterial responses and participating signaling pathways induced by R848 were examined.

## Materials and Methods

### Fish

Golden pompano (body weight 17.2 ± 3.5 g) was purchased from the Haifeng Aquatic Product Company (Hainan Province, China) and kept in indoor aquaria with a flow-through seawater system at 26°C for 1 week before conducting experiments. Animals were anaesthetized with MS-222 before any procedure. Before performing experiments, fish were randomly examined to assure they were free of bacterial infections by plate counting. All animal care in the current study was performed according to the guidelines and regulations and approved by the Animal Care and Use Committee of Hainan University (no. HNU190402, approval date: 3 April 2019).

### Cell Lines

Golden pompano snout (GPS) cells were cultured in L-15 medium (Leibovitz’s L15, Gibco, USA) supplemented with 10% FBS (fetal bovine serum), 1% Pen Strept (penicillin-streptomycin), 0.5% HEPES (1 M), and 1% NaCl in a humidified incubator at 26°C as previously described (35).

### R848 and *Edwardsiella tarda* Infection

To study the antibacterial immunity induced by R848 (InvivoGen, USA) in *T. ovatus*, fish were separated into four groups with 15 fish in each group. Prior to the formal experiment, one preliminary experiment was performed to confirm the best dosage of R848 on golden pompano. Three different dosages (5, 10, and 15 μg per fish) of R848 advised by previous studies were injected intramuscularly (i.m.) into fish, and the result showed that 15 μg of R848 was the most suitable dose (36–38). In the formal experiment, fish were injected i.m. with 100 μl R848 (150 μg/ml), and 100 μl chloroquine (CQ, blocks endosomal acidification) (InvivoGen, USA) (25 μg/ml), 50 μl R848 (300 μg/ml) plus 50 μl CQ (50 μg/ml), and 100 μl PBS (as control), respectively. At 24 h post-stimulation, the fish were injected intraperitoneally (i.p.) with 100 μl of *E. tarda* (5 × 10^6^ CFU/ml) PBMM02142, which was isolated from one diseased *T. ovatus* that was suffered from skin lesions in the Hainan Province of China and saved in our lab, which had been described by Sun et al. (39). At 6, 12, and 24 h post-infection (hpi), the liver, spleen, and kidney were obtained aseptically from five fish randomly selected from each group. The amount of *E. tarda* in these three tissues was counted by the spread plate method. To analyze whether R848 has the bactericidal ability, *in vitro* experiments were conducted. Specifically, R848 was resuspended in PBS at different doses (0, 40, 60, 80, 120, 160, and 320 μg/ml), and 20 μl R848 was incubated with 180 μl of *E. tarda* (10^4^ CFU/ml) for 3 h at 28°C. Then, the number of bacterial colonies was determined by the spread plate method.

### Cell Counting Kit-8 Assay

To evaluate the impact of R848 on head kidney lymphocytes (HKLs) proliferation, HKLs were extracted from three *T. ovatus* (460 ± 54 g) as described previously (40). Subsequently, the HKLs (5 × 10^5^ cells/well) were seeded in a 96-well cell culture plate. Then, the cells were incubated with different doses of R848 (0, 0.175, 0.25, 0.5, 1, 2, 4, 8, 16, and 32 μg/ml) at 26°C for 12 h. The proliferation of HKLs induced by R848 was measured using the Cell Counting Kit-8 (CCK8, Biosharp, China) following the manual. Additionally, HKLs were treated with CQ (final concentration of 5 μg/ml) for 1 h and then treated with R848 (final concentration of 8 μg/ml) at 26°C for 12 h. Afterwards, the proliferation of the HKLs was measured with CCK8 as described above. To examine the impact of Myd88 on the proliferation of cells induced by R848, 50 μM Pepinh-MYD (known to be a Myd88 inhibitory peptide) or 50 μM Pepinh-Control (a control peptide) (InvivoGen, USA) was mixed with HKLs for 6 h at 26°C. Subsequently, the cells were incubated with R848 (final concentration of 8 μg/ml) and the proliferation of the cells was determined as described above. Furthermore, to estimate the impact of NF-κB inactivation on the proliferation of cells induced by R848, 1 μM BAY-11-7082 (Beyotime, China), an IκB-α phosphorylation inhibitor that prevents NF-κB activation, was added to cells and incubated for 1 h at 26°C. Then, R848 (final concentration of 8 μg/ml) was added to the cells, and the proliferation of the cells was detected as described above. All assays were independently performed three times.

### Apoptosis Assay

To evaluate the effect of R848 on HKLs apoptosis, freshly isolated HKLs cultured in 6-well plates (10^6^ cells/well) were treated with CQ (final concentration of 5 μg/ml) for 1 h and then treated with R848 (final concentration of 8 μg/ml) for 24 h; the survival of HKLs was evaluated with the Annexin V-FITC/PI Apoptosis Detection Kit I (BD Pharmingen™, USA) and flow cytometry analysis performed on a Guava easyCyte™ flow cytometer (EMD Millipore Corp., USA). Final data was analyzed with guavaSoft 3.1.1. To examine the requirement for Myd88 in R848-induced HKL apoptosis, 50 μM Pepinh-MYD or Pepinh-Control was incubated with HKLs for 6 h at 26°C. After the incubation, the cells were treated with R848 (final concentration of 8 μg/ml), and flow cytometry was used as described above to detect cell survival. To analyze the impact of NF-κB activation on R848-induced apoptosis in HKLs, 1 μM BAY-11-7082 was added to cells for 1 h at 26°C. Then, the cells were treated with R848 (final concentration of 8 μg/ml). The experiments were repeated in triplicate.

### Luciferase Reporter Assay

To examine the activation of NF-κB, a dual-luciferase reporter assay was performed. GPS cells (1 × 10^6^ cells/well) were cultured in a 24-well plate. Then, 0.3 μg of pGL4.32 (luc2P/NF-κB-RE/Hygro) vector (Promega, USA), which is an NF-κB–specific firefly luciferase reporter vector, and 0.3 μg of pRL-CMV (a control vector) were co-transfected into cells with medium containing 5% FBS using FuGENE^®^ 6 Transfection Reagent Limited Use Label License (Promega, USA) following the manual. At 12 h post-transfection, the GPS cells were cultured in complete medium containing antibiotics and 10% FBS. To evaluate the impact of R848 on the activation of NF-κB, transfected cells were treated with R848 (final concentration of 8 μg/ml), CQ (final concentration of 5 μg/ml), R848 (final concentration of 8 μg/ml) plus CQ (final concentration of 5 μg/ml) or PBS (control) and incubated for 24 h at 26°C. Then, the firefly and *Renilla* luciferase activities of cell lysates were measured with the Dual-Luciferase Reporter Assay Kit (Vazyme, China) according to the manual of GloMax^®^ Discover (Promega, USA). To detect the necessity effect of TLR7 and TLR8 on the NF-κB activation induced by R848, *in vitro* knockdown of TLR7 and TLR8 expression was carried out with small interfering RNA (siRNA) technology using the T7 RiboMAX™ Express RNAi System (Promega, USA) as we previously described (41). Briefly, siTLR7 was synthesized using siTLR7-P1/siTLR7-P2 and siTLR7-P3/siTLR7-P4 (**Table S1**), similar to siTroTLR8 and siRNA-C (control siRNA). GPS cells (1 × 10^6^ cells/well) were seeded in a 24-well plate and co-transfected with pGL4.32, pRL-CMV, and siRNA (siTLR7, siTLR8, siTLR7 plus siTLR8, siRNA-C, or PBS). A 12 h post-transfection, the cells were treated with R848 (final concentration of 8 μg/ml) for 24 h and then evaluated with the dual-luciferase reporter assay described above. To examine the requirement for Myd88 in the NF-κB activity induced by R848, 50 μM Pepinh-MYD or Pepinh-Control was incubated with transfected cells, which were co-transfected with pGL4.32 and pRL-CMV, for 6 h at 26°C. Subsequently, the cells were treated with R848 (final concentration of 8 μg/ml) for 24 h and then evaluated with the dual-luciferase reporter assay. Each experiment was conducted independently three times.

### TLR7/8 Knockdown and *Edwardsiella tarda* Infection *In Vivo*

To evaluate the effects of TLR7 and TLR8 on antibacterial immunity induced by R848 in golden pompano, 75 fish were divided into five groups (15 fish/group) and intramuscularly injected with 15 μg of siRNA-C, siTLR7, siTLR8, siTLR7 plus siTLR8, or PBS. Twelve hours after the stimulation, the fish were treated with 15 μg of R848 *via* intramuscular injection, and another 15 fish were injected with PBS as a control. Then, 24 h later, all the fish were injected intraperitoneally with 100 μl of *E. tarda* (5 × 10^6^ CFU/ml). At 6, 12, and 24 hpi, the bacterial loads in the liver, spleen, and kidney of five fish per group at each time point were counted by plate counting.

### Myd88 Knockdown and *Edwardsiella tarda* Infection *In Vivo*

To examine whether the antibacterial effects of R848 were mediated *via* Myd88, 60 fish were included in this experiment. siMyd88 was synthesized using siMyd88-P1/siMyd88-P2 and siMyd88-P3/siMyd88-P4 (**Table S1**) as described above. Forty-five fish were divided into three groups (15 fish/group) and intramuscularly injected with 15 μg of siRNA-C, 15 μg of siMyd88, or PBS. Twelve hours after the stimulation, some fish were treated with 100 μl R848 (150 μg/ml) *via* intramuscular injection, and another 15 fish were injected with PBS as a control. Then, 24 h later, all the fish were injected intraperitoneally with 100 μl of *E. tarda* (5 × 10^6^ CFU/ml). At 6, 12, and 24 hpi, the liver, spleen, and kidney of five fish per group were obtained aseptically. The bacterial loads in these three tissues were counted by the spread plate method as described above.

### Quantitative Real-Time Reverse Transcriptase-PCR (qRT-PCR) Analysis of the Expression of Immune Genes

To further investigate the effects of TLR7/8 and Myd88 on R848-induced immunity, adaptor genes and some inflammatory genes, including TLR7, TLR8, Myd88, IFN-γ (interferon γ), IRF3 (interferon regulating factor 3), TNF-α (tumor necrosis factor α), interleukin-10 (IL-10), CCL4 [chemokine (C-C motif) ligand 4], and BLyS (B lymphocyte stimulating factor), were examined (**Table S1**). Thirty golden pompanos were injected intramuscularly with siRNA-C, siTLR7, siTLR8, siTLR7 plus siTLR8, siMyd88, or PBS (five fish/group). At 12 h post-administration, some fish were treated with 100 μl R848 (150 μg/ml) *via* intramuscular injection, while another five fish were injected with PBS as a control. Twenty-four hours later, the head kidney was harvested from each fish. Total RNA extraction and cDNA synthesis were performed as described in our previous study (34). qRT-PCR was carried out using the Eastep^®^ qPCR Master Mix Kit (Promega, Madison, USA) according to the manufacturer’s protocol using the primers listed in **Table S1**. The mRNA levels of target genes were determined by the 2^-ΔΔCt^ method with *beta-2-microglobulin* (*B2M*) used as the housekeeping gene according to our previous study (42).

### Statistical Analysis

GraphPad Prism software (version 8, La Jolla CA, USA) was used to analyze data. All quantitative data were analyzed by a t-test. Differences were considered significant with a P-value less than 0.05.

## Results

### Antibacterial Activity of R848 in Golden Pompano

**Figure 1:**
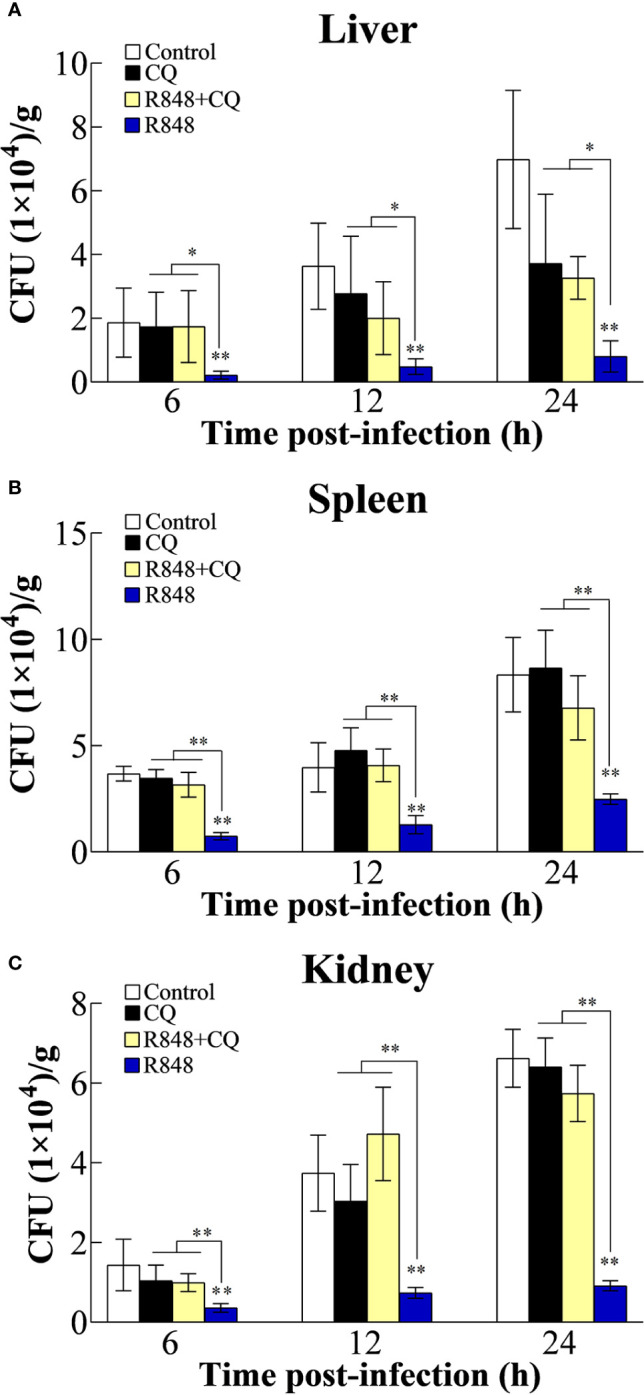
Effect of R848 and chloroquine (CQ) on bacterial infection. Golden pompano administered with R848, CQ, R848 plus CQ, or PBS (control) were infected with *Edwardsiella tarda*, and the amount of bacteria in the liver **(A)**, spleen **(B)**, and kidney **(C)** were determined at different time points. All data are shown as the means ± SD (N = 5), and the statistical significance is indicated. *P < 0.05, **P < 0.01.

To examine whether R848 has antibacterial ability, different doses of R848 were incubated with *E. tarda*. The results showed that the number of bacteria incubated with R848 was similar to that incubated with the control, suggesting that R848 did not have direct bactericidal effects (data not shown). To examine whether R848 can induce antibacterial immunity in golden pompano, fish were injected with R848 with or without CQ prior to infection with *E. tarda*. At 6, 12, and 24 hpi, the bacterial loads in the liver, spleen, and kidney were counted. The results revealed that in all the examined tissues, *E. tarda* infection was extremely significantly decreased in the fish treated with R848 compared to the fish in the control group at each sampling time point. In contrast, the bacterial loads in the fish administered CQ alone or R848 and CQ together were not significantly different from those in the fish in the control group but were significantly higher than those in the R848-treated fish (**Figure 1**).

### Effects of R848 on the Proliferation and Apoptosis of Lymphocytes

#### Effect on Proliferation

**Figure 2:**
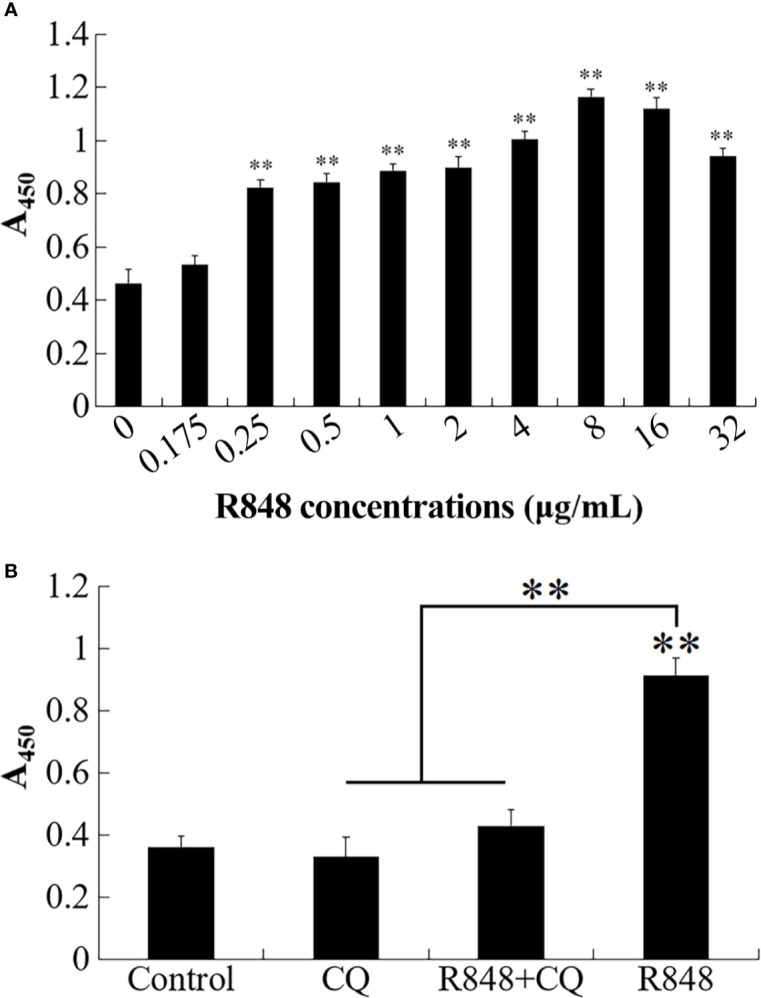
Effect of R848 on the proliferation of HKLs. **(A)** Golden pompano head kidney lymphocytes (HKLs) were treated with different concentrations of R848, and then the proliferation of HKLs was examined by CCK8 assay. **(B)** Golden pompano HKLs were treated with or without (control) 5 µg/ml of chloroquine (CQ), 8 µg/ml R848 plus 5 µg/ml of CQ, or 8 µg/ml R848, and then examined for proliferation by CCK8 assay. Data are presented as means ± SEM (N = 3). N, the number of times the experiment was performed. **P < 0.01.

To evaluate whether lymphocyte proliferation can be enhanced by R848, different doses of R848 were used to treat HKLs derived from golden pompano. CCK8 assay results showed that 0.25–32 μg/ml R848 significantly promoted HKL proliferation (**Figure 2A**). Once CQ was added, the promotive effect of R848 disappeared (**Figure 2B**).

#### Effect on Apoptosis

**Figure 3:**
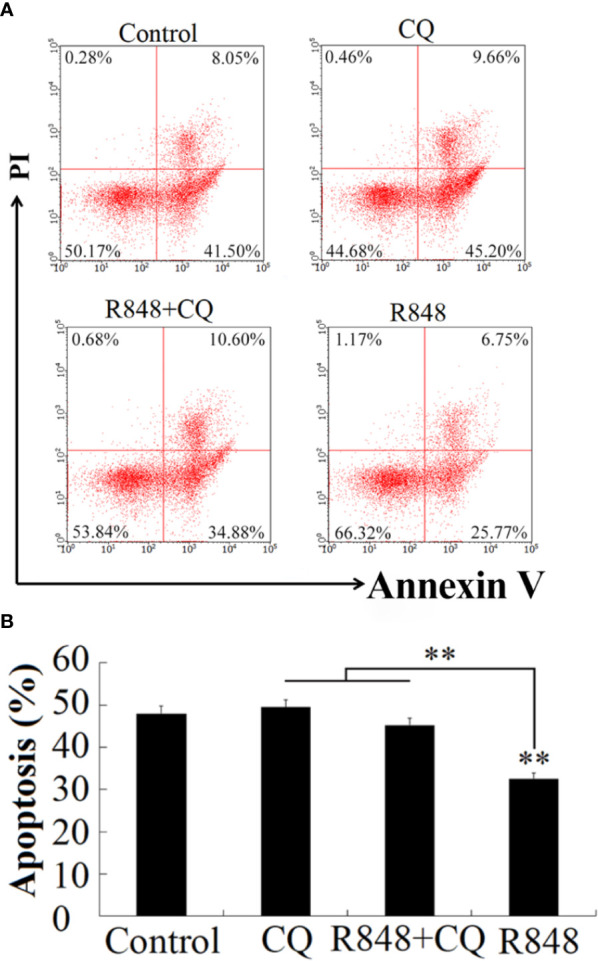
Effect of R848 and chloroquine (CQ) on the apoptosis of HKLs. **(A)** Golden pompano HKLs treated with or without (control) 8 µg/ml R848, 5 µg/ml CQ, or 8 µg/ml R848 plus 5 µg/ml CQ, and then the apoptosis rates of these cells were determined by the flow cytometry. **(B)** The percentages of apoptotic cells are represented with statistic bar charts. Data are presented as means ± SD (N = 3). N, the number of times the experiment was performed. **P < 0.01.

To detect whether R848 can induce HKL apoptosis, an Annexin V-FITC/PI staining assay was performed. After R848 monotherapy or combination treatment with R848 and CQ for 24 h, cellular apoptosis was evaluated. The results suggested that the number of apoptotic cells was significantly lower with R848 monotherapy (32.52%) than with control treatment. However, treatment with R848 and CQ together resulted in a significantly higher number of apoptotic cells (45.48%) than treatment with R848 alone, but there was no significant difference compared with control treatment (49.55%) (**Figure 3**).

### Myd88-Dependent Immune Responses Induced by R848

**Figure 4:**
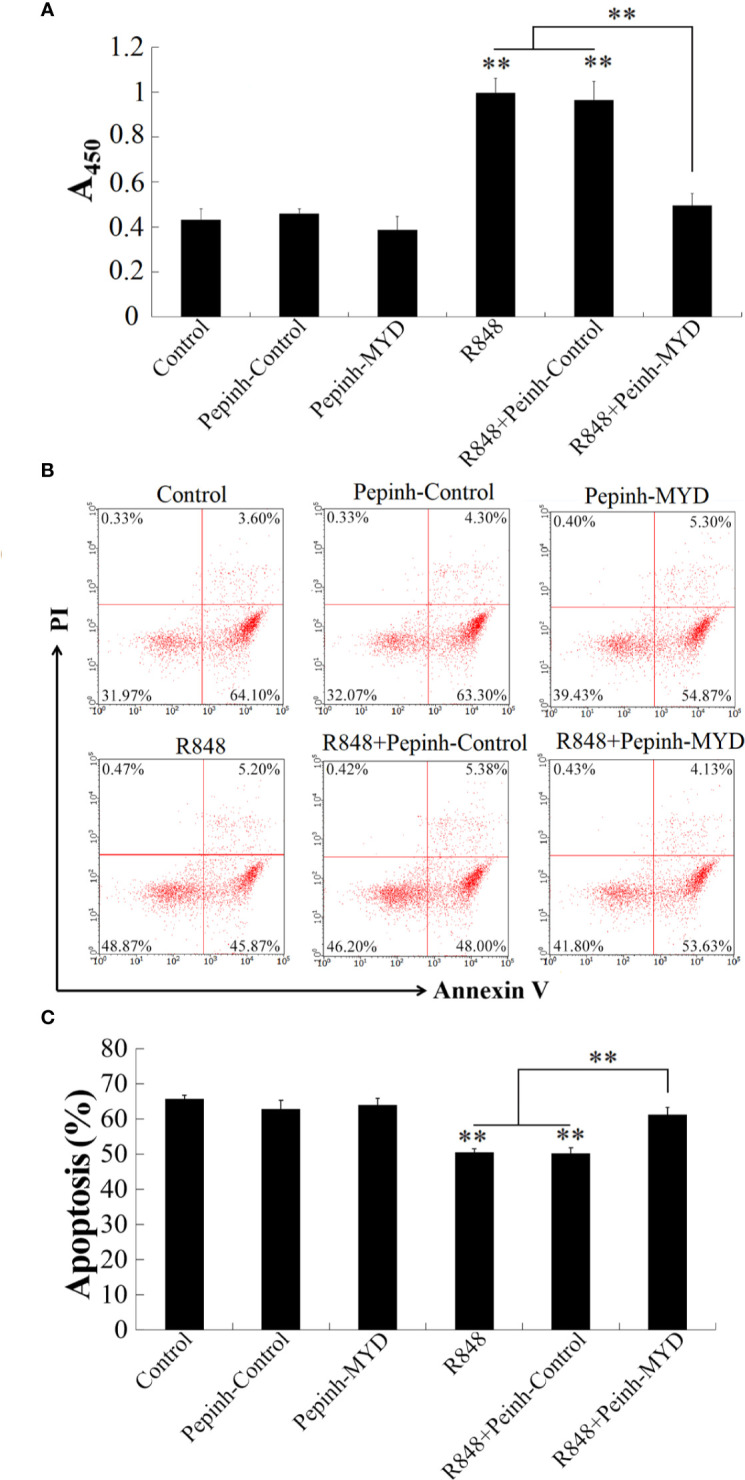
R848-induced cellular activity through Myd88-signaling pathway. Golden pompano HKLs were treated with PBS (control), R848, Pepinh-Control, Pepinh-MYD, R848 plus Pepinh-Control, or R848 plus Pepinh-MYD. The cells were then determined for proliferation **(A)**, apoptosis **(B)**, and statistic bar chart of the apoptosis percentage **(C)**. Data in are presented as means ± SEM (N = 3), and **(C)** are presented as means ± SD (N = 3). **P < 0.01. Data in **(B)** are one representative of three independent experiments.

To explore whether Myd88 is involved in R848-induced immunity, the effect of Pepinh-MYD (Myd88 inhibitor) on the proliferation of HKLs induced by R848 was determined. The results showed that the proliferation of HKLs treated with R848 and Pepinh-MYD together was significantly decreased compared with that of cells stimulated with R848 with or without Pepinh-Control, whereas there was no significant difference between the cells administered R848 and those administered R848 plus Pepinh-Control. In contrast, cells stimulated with Pepinh-MYD or R848 plus Pepinh-MYD showed no significant differences from control cells (**Figure 4A**). In line with these results, Annexin V-FITC/PI staining revealed that R848 significantly inhibited apoptosis in HKLs compared to PBS (control), but the effect was weakened when Pepinh-MYD but not Pepinh-Control was present (**Figures 4B, C**).

### NF-κB Activity Induced by R848 and Its TLR7/8 and Myd88 Dependence

**Figure 5:**
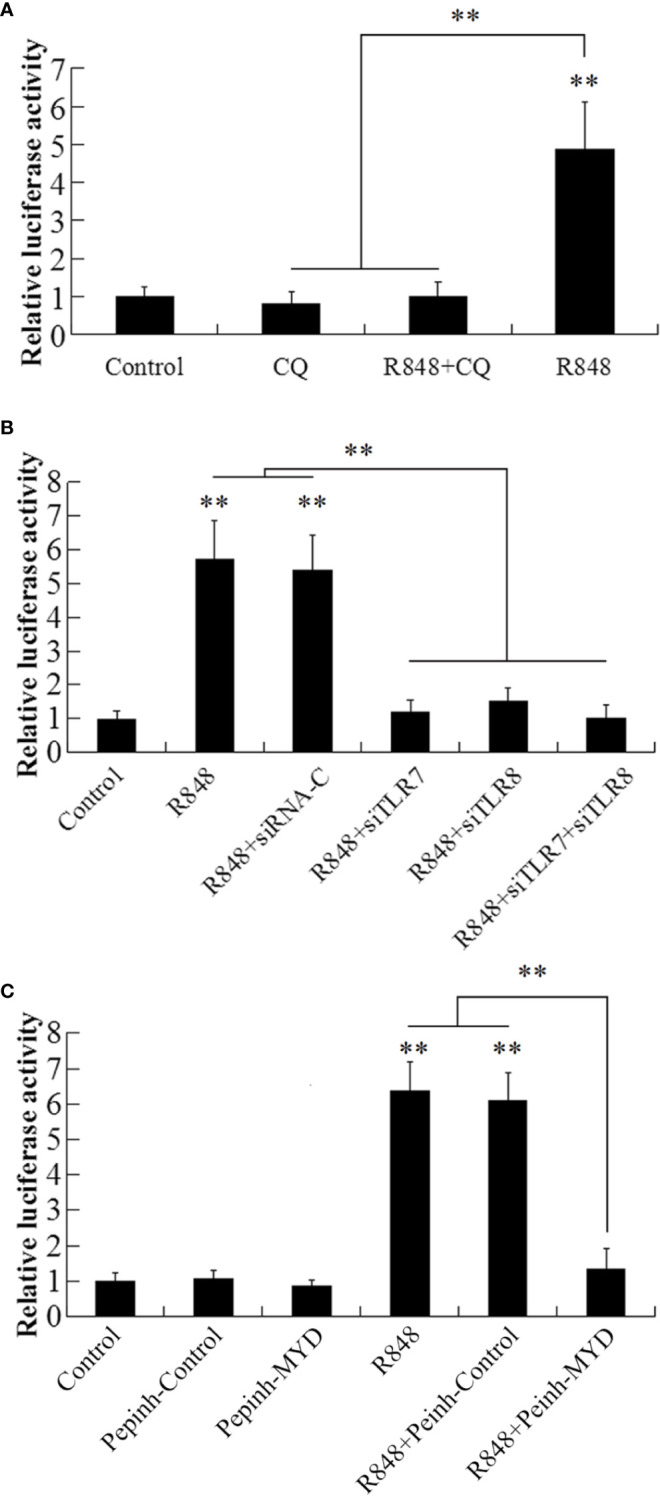
Effect of R848 on NF-κB activity. GPS cells containing NF-κB-RE-*luc2P* reporter were treated with or without (control) R848, chloroquine (CQ), or R848 plus CQ and then analyzed for luciferase activity **(A)**. GPS cells containing NF-κB-RE-*luc2P* reporter were transfected with siTLR7, siTLR8, siTLR7 plus siTLR8, siRNA-C, or PBS (control) and then treated with R848. The luciferase activity in the cells was then determined **(B)**. GPS cells containing NF-κB-RE-*luc2P* reporter were treated with R848 in the presence or absence of Pepinh-Control or Pepinh-MYD; the control cells were treated with PBS. The luciferase activity in the cells was then determined **(C)**. Data are expressed as means ± SEM (N = 3). N, the number of times the experiment was performed. **P < 0.01.

To detect the NF-κB activity induced by R848, GPS cells were transfected with NF-κB-RE-*luc2P*, and then the cells were stimulated with R848 with or without CQ. The results demonstrated that the luciferase activity of the cells stimulated with R848 was significantly increased (4.9-fold) compared to that of the control cells, whereas the luciferase activity of the cells stimulated with R848 and CQ together was not significantly different from that of the control cells (**Figure 5A**). When TLR7 and TLR8 expression was knocked down by transfection with siTLR7 and siTLR8, respectively, luciferase activity was significantly decreased compared with that stimulated with R848 alone or R848 plus siRNA-C. Furthermore, when siTLR7 and siTLR8 were transfected together, the decrease in luciferase activity was enhanced (**Figure 5B**). When GPS cells were treated with R848 and Pepinh-MYD, significantly decreased luciferase activity was observed compared with that observed when cells were administered R848 alone or R848 plus Pepinh-Control (**Figure 5C**).

### NF-κB-Dependent Signaling in R848-Induced Immune Response

**Figure 6:**
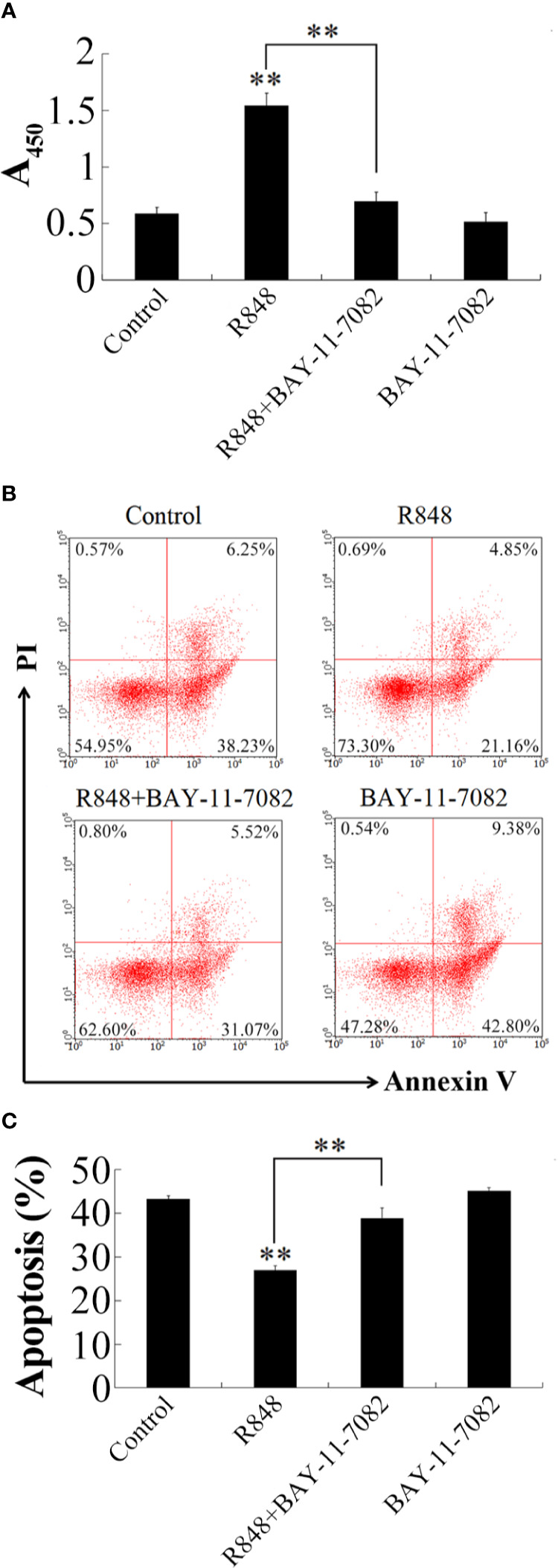
Effect of BAY-11-7082 on R848-induced cellular activity. **(A)** Golden pompano HKLs were treated with PBS (control), R848, BAY-11-7082, or R848 plus BAY-11-7082. The cells were then determined for proliferation **(A)**, apoptosis **(B)**, and statistic bar chart of the apoptosis percentage **(C)**. Data in **(A)** are presented as means ± SEM (N = 3), and **(C)** are presented as means ± SD (N = 3). **P < 0.01. Data in **(B)** are one representative of three independent experiments.

To further detect the immune response induced by R848 whether relying on NF-κB, HKLs were incubated with R848 with or without BAY-11-7082 (NF-κB inhibitor), and then cell proliferation and apoptosis were examined. The results showed that in the cells treated with R848 and BAY-11-7082 together, the effects of R848 were significantly reduced compared with those in cells treated with R848 alone (**Figure 6A**). Correspondingly, Annexin V-FITC/PI staining revealed that apoptosis was significantly reduced in HKLs treated with R848 (26.01%) compared to control cells (44.48%). However, the apoptosis rate of HKLs stimulated with R848 and BAY-11-7082 together demonstrated a significant increase compared with that of the cells stimulated with R848 alone but was comparable to that of the control cells (**Figures 6B, C**).

### *In Vivo* Antibacterial Effect of R848 Mediated Through the TLR7/8-Myd88-Dependent Pathway

#### Effects of TLR7/8 and Myd88 Knockdown on Bacterial Infection

**Figure 7:**
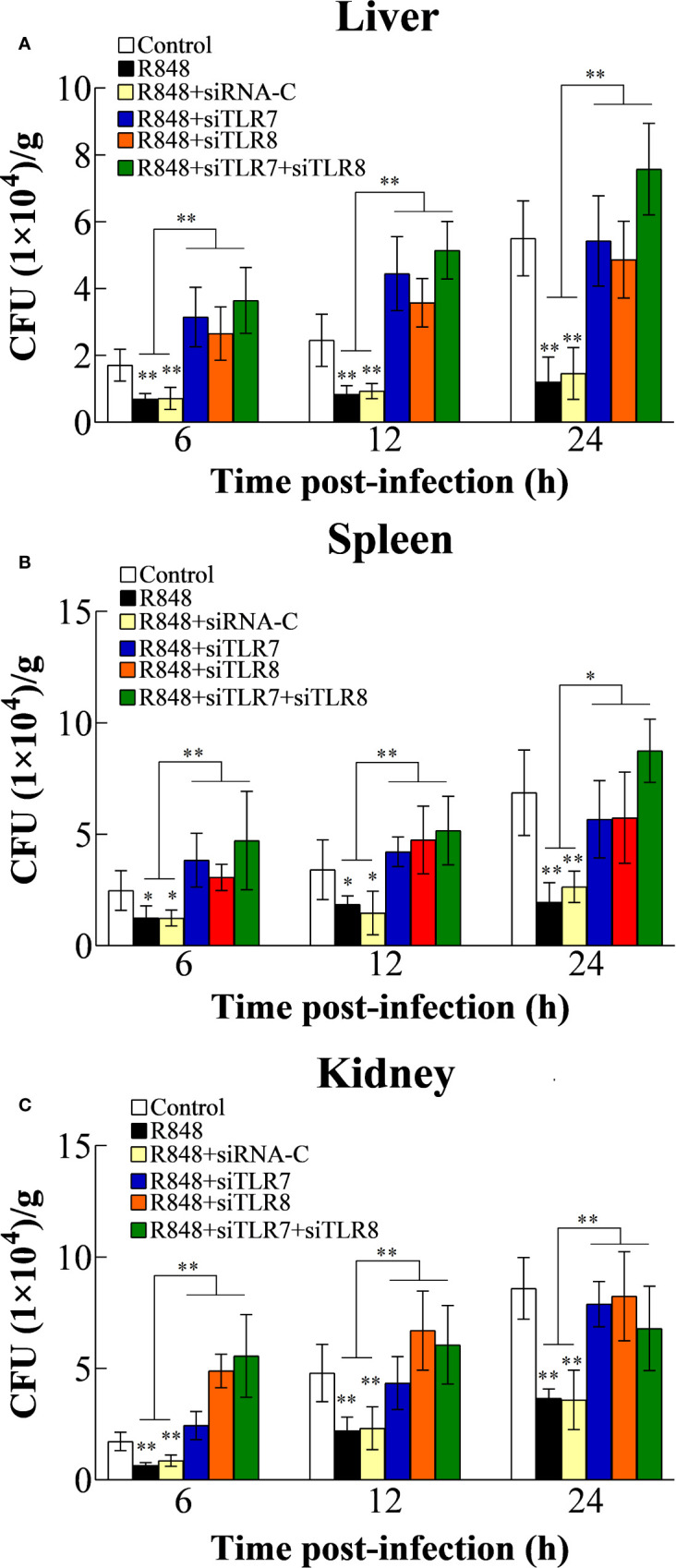
Effect of R848 on bacterial infection through TLR7/8-signaling pathway. Golden pompano administered with R848, R848 plus siRNA-C, R848 plus siTLR7, R848 plus siTLR8, R848 plus siTLR7 and siTLR8, or PBS (control) were infected with *Edwardsiella tarda*, and the amount of bacteria in the liver **(A)**, spleen **(B)**, and kidney **(C)** were determined at different time points. All data are shown as the means ± SD (N = 5), and the statistical significance is indicated. **P < 0.01.

**Figure 8:**
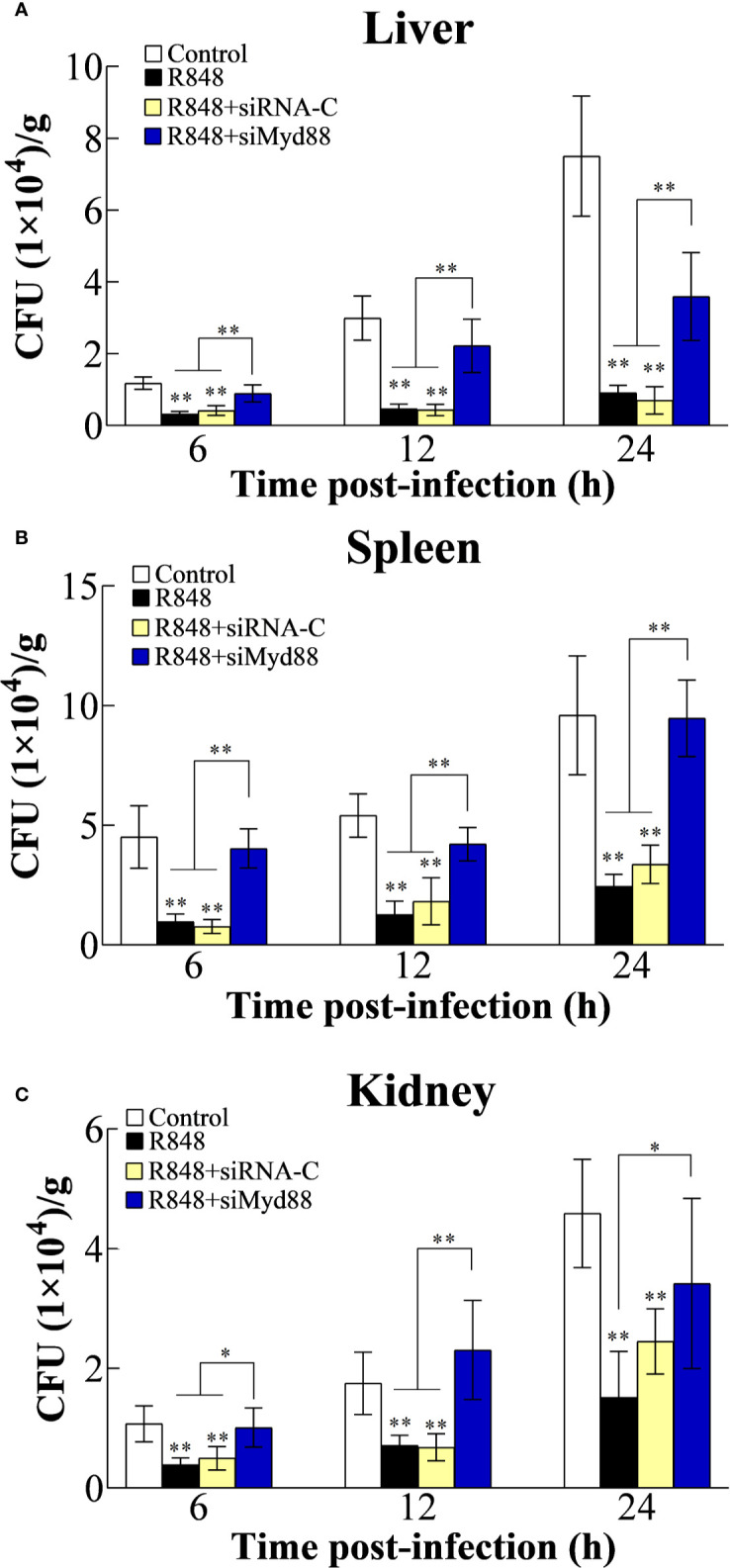
Effect of R848 on bacterial infection through Myd88-signaling pathway. Golden pompano administered with R848, R848 plus siRNA-C, R848 plus siMyd88, or PBS (control) were infected with *Edwardsiella tarda*, and the amount of bacteria in the liver **(A)**, spleen **(B)**, and kidney **(C)** were determined at different time points. All data are shown as the means ± SD (N = 5), and the statistical significance is indicated. *P < 0.05, **P < 0.01.

To further analyze the antibacterial effect induced by R848 *in vivo* and the associated working mechanism, the expression of TLR7/8 and Myd88 was knocked down in golden pompano *via* siRNA technology. For this purpose, golden pompano fish were administered synthesized siRNAs (siTLR7, siTLR8, siTLR7+siTLR8, or siRNA-C) and then stimulated with R848. Our *in vivo* experiments showed that at 6, 12, and 24 hpi, the bacterial amounts in the liver, spleen, and kidney of the fish stimulated with R848 or R848 plus siRNA-C were significantly decreased compared with those of the fish stimulated with PBS (control) or R848 with preinjection of siTLR7, siTLR8, or siTLR7+siTLR8 (**Figure 7**). To further evaluate the significance of Myd88 in the antibacterial response induced by R848, Myd88 expression was knocked down *in vivo via* siRNA technology. For this purpose, golden pompano fish were administered synthesized siRNA (siMyd88 or siRNA-C) and R848. The results suggested that at 6, 12, and 24 hpi, significantly lower numbers of bacterial colonies were found in the liver, spleen, and kidney of fish administered R848 or R848 with preinjection of siRNA-C than those of fish treated with R848 and preinjection of siMyd88 (**Figure 8**).

### Expression of Immune Genes

**Figure 9:**
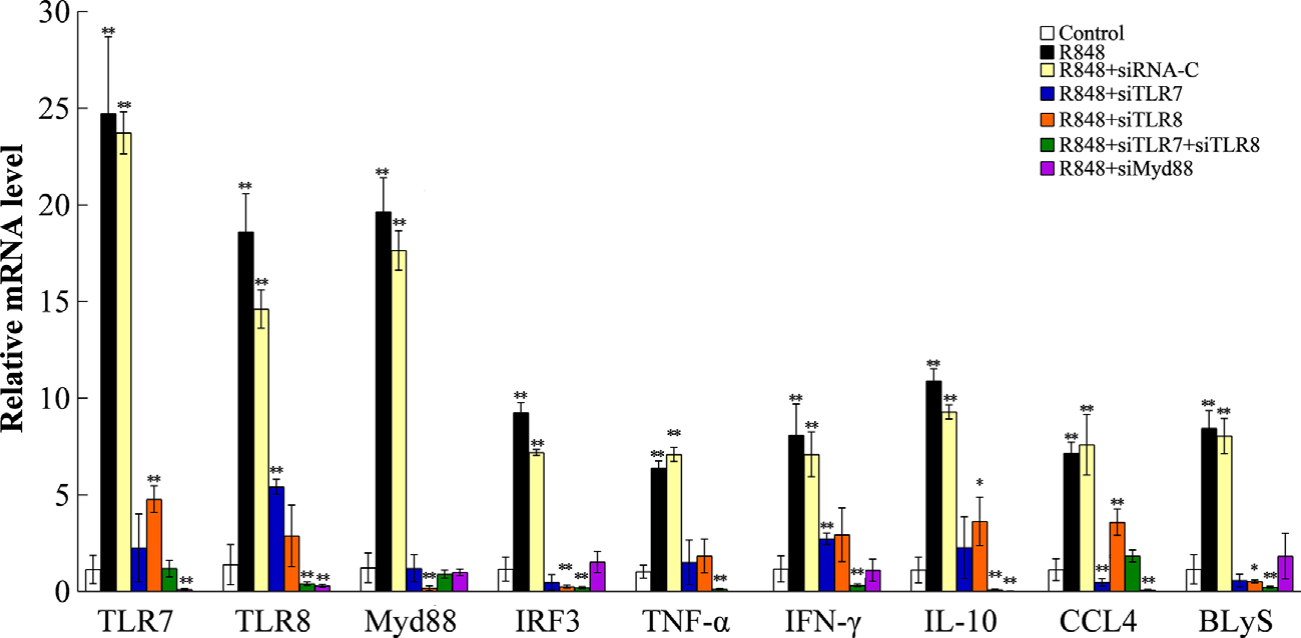
Effect of R848 on the expression of immune genes through TLR7/8-Myd88-signaling pathway. Expression of golden pompano TLR7/8 and Myd88 were knocked down in golden pompano *via* siRNA technology. Then, fish were treated with or without (control) R848. The expression profiles of immune genes were investigated in head kidney by qRT-PCR. The internal reference gene was *B2M*. Values are shown as means ± SD (N=5), and the statistical significance is indicated. *P < 0.05, **P < 0.01.

To understand the working mechanism of R848 *in vivo*, after knocking down the expression of TLR7/8 and Myd88 as described above, we examined the expression of immune-related genes. qRT-PCR was carried out to analyze the expression of the TLR7, TLR8, Myd88, IFN-γ, IRF3, TNF-α, IL-10, CCL4, and BLyS genes, with *B2M* as an internal control. The results showed that R848 significantly increased the expression of all the examined immune-related genes compared to control treatment. R848 plus siRNA-C was comparable to R848. In contrast, after knockdown of TLR7, TLR8, or Myd88 expression, the expression of all the examined immune-related genes were significantly reduced compared with that in the R848 group (**Figure 9**). The effects induced by R848 on the expression of immune-related genes were almost abolished after knockdown of TLR7, TLR8, or Myd88 expression.

## Discussion

R848 is a low-molecular-weight imidazoquinoline compound that can be recognized by TLR7 and TLR8 (1). As effective agents to treat cancer and infectious diseases and to improve immune responses, agonists of TLR7 and TLR8 have attracted great attention. At present, R848 is mainly used in the treatment of infectious viral diseases and tumors (43–45). However, there are few reports on its antibacterial activity in fish. In the current study, we found that R848 could significantly reduce the bacterial loads in the liver, kidney, and spleen of *T. ovatus*, which indicated that R848 induced effective immune responses in lower vertebrates. This study sought to evaluate the mechanism involving R848 and TLR7/8 in fish.

As an analog of imiquimod (R837), R848 is the first imidazoquinoline recognized by both TLR7 and TLR8. The immune responses induced by R848 and R837 are similar; however, the immune effects elicited by R848 are 50–100 times greater than those elicited by R837 (46–48). Many studies have shown that R848 elicits effective immune responses against viral infection (43, 46–49). Some studies have reported that it also has antibacterial properties (44, 50). In fish, studies on R848 have focused on its immunostimulatory and antiviral effects, but its antibacterial effects and working mechanism are still unknown (15, 37, 51–54). In this study, we found that after golden pompano administered with R848, bacterial loads in the liver, spleen, and kidney after *E. tarda* infection were significantly reduced; however, R848 did not have direct antimicrobial activity. These results suggested that R848 induced the immune responses against bacteria in golden pompano.

In mammals, R848 is recognized by human TLR7/8 and mouse TLR7 and then activates many immune cells, such as pDCs, macrophages, and monocytes, leading to the secretion of IFN I, IL-8, IL-12, TNF-α, and so on (8, 16, 55). Previous studies have shown that R848 induces the activation of CD4^+^ Th1 cells and CD8^+^ cytotoxic T lymphocytes (8, 55), promotes the proliferation of B cells and inhibits their apoptosis (21, 56, 57). Recently, R848 was demonstrated to enhance human B cell differentiation induced by IL-21 and sCD40L (58). In mice, R848 augments the proliferation of the bone marrow CD11c^+^ B lymphocyte population (59). R848 increases the proliferation of lymph node cells and promotes B cell activation (60). Consistent with these observations, R848 was found to increase the production of BlyS, which could promote the proliferation, differentiation, and survival of B cells (61). In addition, R848 was found to induce a cytotoxic T lymphocyte (CTL) response and maturation of monocytes (45). In line with this, we found that R848 promoted HKLs proliferation and reduced HKLs apoptosis. Further studies showed that when TLR signaling was blocked with CQ (62, 63), the effects induced by R848 were significantly reduced, suggesting that R848 may act through a TLR signaling pathway.

TLR7 and TLR8 in golden pompano were reported to exhibit the typical TLR domain architecture, including a TIR domain located in the cytoplasmic region (64). When TLRs recognize PAMPs, the TLR conformation changes, and then adaptor molecules, including Myd88, are recruited (65, 66). Previous studies have shown that Myd88 is required for activation of the TLR7/8 pathway. The TIR domains of Myd88 and TLR7/8 interact and then recruit interleukin receptor-associated kinases (IRAKs), including IRAK1, IRAK2, IRAK4, and IRAK-M (19). Once IRAK4 is phosphorylated, it dissociates from Myd88 molecules and interacts with TRAF6 to activate the NF-κB pathway and induce the secretion of proinflammatory cytokines (14, 21). Studies have demonstrated that the immune responses induced by R848 are abolished in TLR7- and Myd88-deficient mice and that the production of inflammatory cytokines is inhibited, suggesting that R848 functions through the TLR7/TLR8 Myd88-dependent signaling pathway (21, 67, 68). In this study, our results showed that when Myd88 activation was blocked by Pepinh-MYD, the proliferation and apoptosis of HKLs were comparable to those of control-treated cells, which demonstrated that Pepinh-MYD significantly reduced the effects of R848, suggesting that Myd88 is required in this immune response in teleosts.

Previous studies have shown that Toll-like receptor ligands promote multiple myeloma cell growth and survival *via* activation of NF-κB (69). NF-κB is also known to play key roles in the inflammatory response and immune response. Normally, NF-κB binds to members of an inhibitory protein family, mainly in the form of the p65/P50/I-κB complex (70, 71). NF-κB activation is induced by various stimuli, such as bacteria, viruses, and cytokines (72–75). TLR-NF-κB signal transduction transmits activation signals to cells through a series of adaptor proteins, thus activating NF-κB to regulate the expression of related genes, especially those encoding inflammatory cytokines. *In vitro* transfection experiments previously showed that R848 induces the activation of NF-κB in HEK293 cells in a dose-dependent manner (21). Consistent with this, many studies have shown that NF-κB is needed for the production of cytokines in mammalian cells treated with R848 (76, 77). In line with these findings, our results demonstrated that R848 could significantly induce the activation of NF-κB, but when CQ was added, NF-κB activation disappeared. Further studies showed that when TLR7 and TLR8 expression was knocked down in cells, the activation of NF-κB induced by R848 was significantly reduced. When Myd88 was inhibited by Pepinh-MYD, R848-induced NF-κB activation was significantly decreased. Our results indicate that R848 induces the activation of NF-κB through the TLR7/8-Myd88 signaling pathway. In addition, our results showed that NF-κB plays important roles in the immune effects induced by R848. When HKLs were treated with BAY-11-7082, which was used to suppress the activation of NF-κB, the effects of increased cell proliferation and decreased apoptosis induced by R848 were significantly reduced. It is well known that *in vivo* animal models are very useful approaches to study immune responses. In line with our *in vitro* studies, when TLR7, TLR8, and Myd88 expression was knocked down by siRNA following injection of R848 into golden pompano, bacterial invasion in the liver, spleen, and kidney in the context of *E. tarda* infection was enhanced compared to when R848 treatment was administered alone, suggesting that R848-induced antibacterial immunity was significantly reduced. These results further support the conclusion that R848 plays essential roles in the antibacterial immunity of golden pompano *via* the TLR7/8 Myd88-dependent signaling pathway. Previous studies have shown that TLR7/TLR8 recognize R848 and then activate NF-κB *via* a Myd88-dependent signaling pathway, ultimately inducing the secretion of a variety of cytokines, such as IFN-γ, TNF-α, and BlyS, that mediate innate and acquired immunity (61, 67, 68). In line with these findings, our results showed that R848 significantly induced the expression of IFN-γ, TNF-α, IL-10, CCL4, and BlyS. In contrast, when TLR7, TLR8, and Myd88 expression was knocked down by siRNA, this increased expression was reduced, suggesting that TLR7, TLR8, and Myd88 are required for the R848-induced effects.

In conclusion, we illustrated that R848 is an immunostimulatory molecule using golden pompano as a model and that R848 enhanced the resistance to bacterial infection and induced immune responses. Moreover, the antibacterial effects of R848 might be mediated *via* the TLR7/8-Myd88-NF-κB signaling pathway.

## Data Availability Statement

The raw data supporting the conclusions of this article will be made available by the authors, without undue reservation.

## Ethics Statement

The animal study was reviewed and approved by the Animal Care and Use Committee of the Hainan University.

## Author Contributions

YZ, XC, and YS designed the research. ZC, JL, and HL analyzed the data. YW and ZZ supervised the study. YZ and XC performed the research, analyzed the data, and wrote the manuscript with support from all authors. All authors contributed to the article and approved the submitted version.

## Funding

This research was funded by the National Natural Science Foundation of China (No. 41666006 and No. 42066007), and the Nanhai Famous Youth Project.

## Conflict of Interest

The authors declare that the research was conducted in the absence of any commercial or financial relationships that could be construed as a potential conflict of interest.
