# Supplementary material for: R848 Is Involved in the Antibacterial Immune Response of Golden Pompano (Trachinotus ovatus) Through TLR7/8-MyD88-NF-κB-Signaling Pathway
Source: Front Immunol. 2021 Jan 18;11:617522. doi: 10.3389/fimmu.2020.617522 (PMC7848160; doi:10.3389/fimmu.2020.617522)
Supplement: Supplementary file 1 [file Table_1.doc]

Table S1 Primers used in this study

| Primer name | Primer sequences (5’-3’) |  |
| --- | --- | --- |
| siRNA-C-P1 | GGATCCTAATACGACTCACTATAGCCACAACGTCTATATCAT | Synthesizing siRNA-C |
| siRNA-C-P2 | AAATGATATAGACGTTGTGGCTATAGTGAGTCGTATTAGGATCC |  |
| siRNA-C-P3 | GGATCCTAATACGACTCACTATAATGATATAGACGTTGTGGC |  |
| siRNA-C-P4 | AAGCCACAACGTCTATATCATTATAGTGAGTCGTATTAGGATCC |  |
| siTLR7-P1 | GGATCCTAATACGACTCACTATAGCTGCTGCTGAGTACCAAA | Synthesizing siTLR7 |
| siTLR7-P2 | AATTTGGTACTCAGCAGCAGCTATAGTGAGTCGTATTAGGATCC |  |
| siTLR7-P3 | GGATCCTAATACGACTCACTATATTTGGTACTCAGCAGCAGC |  |
| siTLR7-P4 | AAGCTGCTGCTGAGTACCAAATATAGTGAGTCGTATTAGGATCC |  |
| siTLR8-P1 | GGATCCTAATACGACTCACTATAGCTTGAGACACTACACCTA | Synthesizing siTLR8 |
| siTLR8-P2： | AATAGGTGTAGTGTCTCAAGCTATAGTGAGTCGTATTAGGATCC |  |
| siTLR8-P3： | GGATCCTAATACGACTCACTATATAGGTGTAGTGTCTCAAGC |  |
| siTLR8-P4： | AAGCTTGAGACACTACACCTATATAGTGAGTCGTATTAGGATCC |  |
| siMyd88-P1 | GGATCCTAATACGACTCACTATA GGATGTCAAGAGGTTTCAT | Synthesizing siMyd88 |
| siMyd88-P2 | AAATGAAACCTCTTGACATCCTATAGTGAGTCGTATTAGGATCC |  |
| siMyd88-P3 | GGATCCTAATACGACTCACTATAATGAAACCTCTTGACATCC |  |
| siMyd88-P4 | AAGGATGTCAAGAGGTTTCATTATAGTGAGTCGTATTAGGATCC |  |
| B2M-F1 | AAGTCAGTCCACCCAAGGTTCA | qPCR |
| B2M-R1 | GGGATTTCCATTCCGTTCTTCATG |  |
| TLR7-F | GCTCAACAGGACCACAGTGACC | qPCR |
| TLR7-R | GATTACAGGATGACCTATTTGCG |  |
| TLR8-F | TGGGTGATGAGAAATCTGCG | qPCR |
| TLR8-R | GCCTCTGTTAAGACAAAAAGGG |  |
| Myd88-F1 | GACGGTGGGAAAGTTGTTGTC | qPCR |
| Myd88-R1 | GGAAGTCACTCTGGCAGTAGCA |  |
| IRF3-R1 | TCGCACGAACTCCTTCAACAT | qPCR |
| IRF3-F1 | TCAAGCCAATCTGACCAAACG |  |
| TNF-α-F | GGCGTCGTTCAGAGTCTCCT | qPCR |
| TNF-α-R | TCCTCCTGGGCAGTGGTTT |  |
| IFN-γ-F | GTTGGAAGTGGGCGAGGAT | qPCR |
| IFN-γ-R | TTTGTTCTGGACGACGAGGTT |  |
| IL-10-F | AGTCAGTCTCCACCCCCATCTT | qPCR |
| IL-10-R | GCCCACTGGAGTTCAGATGCT |  |
| CCL4-F | TTTGTGCTGATGCTGGCTTTC | qPCR |
| CCL4-R | CCGCTGGCTGGTCTTGATG |  |
| BLyS- F | TGGCAGACGGGACTAAGGA | qPCR |
| BLyS-R | TGTTGAAAGGGAAGACAGGGTT |  |
